# Physiological and Transcriptome Analyses Provide Insights into the Response of Grain Filling to High Temperature in Male-Sterile Wheat (*Triticum aestivum* L.) Lines

**DOI:** 10.3390/ijms252212230

**Published:** 2024-11-14

**Authors:** Qiling Hou, Jiangang Gao, Hanxia Wang, Zhilie Qin, Hui Sun, Shaohua Yuan, Yulong Liang, Changhua Wang, Fengting Zhang, Weibing Yang

**Affiliations:** Institute of Hybrid Wheat, Beijing Academy of Agricultural and Forestry Sciences, Beijing 100097, China; wheatqilinghou@163.com (Q.H.); gjg86520@126.com (J.G.); wanghanxia314@sina.com (H.W.); qinzhl_bj@126.com (Z.Q.); sunhui628@sina.com (H.S.); yuanshaohua@baafs.net.cn (S.Y.); yulong_liang@163.com (Y.L.); wangchanghua78@sohu.com (C.W.); lyezh@163.com (F.Z.)

**Keywords:** high temperature, gene expression, sucrose unloading, starch synthesis, grain filling

## Abstract

High-temperature (HT) stress frequently affects the early and middle stages of grain filling in hybrid seed production regions. Photo-thermo-sensitive male-sterile (PTMS) wheat lines, which play a critical role as female parents in hybrid seed production, face challenges under HT conditions. However, the mechanisms governing grain filling in PTMS lines under HT stress remain poorly understood. This study used the BS253 line to investigate the effects of HT on grain filling, primarily focusing on the transition from sucrose unloading to starch synthesis. The findings indicated that HT significantly reduced the grain starch content and weight by 7.65% and 36.35% at maturity, respectively. Further analysis revealed that the expression levels of *TaSUT1* and *TaSWEETs* in grains initially increased after HT stress, paralleling the rise in sucrose content during the same period. The activities of ADP-glucose pyrophosphorylase, UDP-glucose pyrophosphorylase, granule-bound starch synthase, and soluble starch synthase were markedly decreased, indicating that impaired starch synthesis was a key factor limiting grain filling immediately after HT exposure. A total of 41 key regulatory genes involved in sucrose-to-starch metabolism were identified, with HT significantly reducing the expression of genes associated with pathways from sucrose unloading to starch synthesis during the middle and late stages post-HT. Based on the observed ultrastructural changes in the abdominal phloem and sucrose transporter expression levels under HT, we concluded that limited sucrose supply, degradation, and inhibition of starch synthesis collectively constrained grain filling during these stages. Additionally, 11 heat shock proteins and two catalase genes were identified and significantly upregulated during the initial phase post-HT, suggesting their potential role in enhancing sucrose supply at this critical time. More importantly, seven key genes involved in the sucrose-to-starch pathway were identified by weighted gene co-expression network analysis (*WGCNA*), which provides target genes for their functional research for starch synthase. These findings provide a comprehensive understanding of how HT limits grain filling, identify several genes involved in the sucrose-to-starch pathway, and offer a novel perspective for future research on HT-restricted grain filling across the entire process from sucrose unloading to starch synthesis in developing grains.

## 1. Introduction

Wheat (*Triticum aestivum* L.) is among the most widely cultivated cereal crops globally [1]. Under current climatic conditions, high-temperature (HT) stress presents a substantial challenge to wheat growth and productivity [1,2]. In particular, HT stress affects crop source–sink relationships during grain filling by accelerating phenological development and inducing premature leaf senescence, which ultimately reduces grain yield [3,4,5]. The extent of yield reduction is associated with both the duration of HT exposure and the developmental stage at which it occurs [6]. The grain-filling stage is considered most sensitive to HT stress [7,8,9]. Exposure to temperatures between 32 °C and 38 °C during the grain-filling stage can reduce yield potential by approximately 50% [10], likely due to disruptions in endosperm cell cellularization and rapid dry matter accumulation in grains [11,12]. HT affects grain filling throughout the process, from the supply of photosynthetic assimilates to starch synthesis. Previous studies have indicated that oxidative damage to chloroplasts, reduced CO_2_-exchange rates, and disruption of the photosystem II complex contribute to decreased photosynthetic capacity in leaves [13,14]. In addition, HT reduces grain yield by impairing starch biosynthesis and limiting the movement of assimilates during grain filling stages [15]. The response of wheat to HT is complex and regulated by numerous genes [12]. Recent transcriptome studies have provided insights into molecular mechanisms underlying wheat’s responses to HT during grain filling [14,16,17]. In addition, the roles of heat shock proteins (HSPs), phytohormones (e.g., abscisic acid and ethylene), and noncoding RNAs in response to HT stress have been extensively documented [9,14,18].

Grain filling involves several processes, including the remobilization of photoassimilates and their transport to storage organs [19]. This transport is mediated by proteins such as sucrose transporters (SUTs) and bidirectional sugar transporters (SWEETs) [20,21]. The phloem, which consists of sieve elements (SEs), companion cells (CCs), intermediary cells (ICs), and phloem parenchyma cells (PPCs), serves as the conduit between source and sink tissues, playing a critical role in the movement of sucrose [22]. Once unloaded into the grains, sucrose is hydrolyzed by sucrose synthases (SuS) and invertases (INVs) into monomer sugars, including glucose/UDP-glucose and fructose, which are subsequently converted into glucose-1-phosphate [23]. Glucose-1-phosphate is then used to synthesize starch through the catalytic action of enzymes, such as ADP-glucose pyrophosphorylase (AGP), granule-bound starch synthase (GBSS), starch synthase (SS), and starch branching enzymes (SBE) [24]. Previous studies have demonstrated that HT can reduce the expression of key genes involved in the starch synthesis pathway, thereby impairing starch accumulation during grain filling [25].

Hybrid wheat is expected to play a crucial role in meeting the demands of a growing population [26,27]. In China, the technical system for two-line hybrid wheat has been established and is currently undergoing rapid development [28,29]. Photo-thermo-sensitive male-sterile (PTMS) lines are essential components of the two-line system, exhibiting high sterility in hybrid seed production regions (approximately 33° N) and restored fertility in reproductive areas (approximately 39° N). The optimal temperature for wheat anthesis and grain filling ranges from 12 °C to 22 °C [2]. However, HTs surpassing 30 °C frequently occur during the mid-grain-filling stage in wheat hybrid seed production areas, with these conditions persisting for 15–20 days, leading to poor grain plumpness and decreased seed vitality. However, research on how grain filling responds to HT, particularly considering both the entire grain-filling stage and the sucrose-to-starch metabolic pathway, remains limited. PTMS lines in wheat hybrid seed production areas typically flower 4 to 5 days earlier than restorer lines. This early flowering, however, delays pollination, which in turn postpones the development of outcrossing grains, complicating the study of sucrose unloading and transport processes in PTMS lines. Both sucrose unloading and transport in outcrossing and self-pollinated grains occur within maternal tissues [7,30]. In reproductive areas, PTMS lines undergo immediate pollination upon flowering, thus overcoming the issue of delayed pollination. Therefore, conducting experiments on HT stress in regions where PTMS lines are cultivated is more feasible and can provide a theoretical basis for understanding outcrossing grain-filling responses to HT. In this study, we systematically investigated the regulatory mechanisms of HT stress in PTMS lines throughout the grain-filling period, focusing on identifying key regulatory genes involved in the sucrose unloading and starch synthesis pathways. Our findings will help bridge the knowledge gap in understanding sucrose unloading and starch synthesis in PTMS lines, provide target genes for enhancing grain filling in PTMS lines, and establish a foundation for further research into outcrossing grain filling under HT stress conditions.

## 2. Results

### 2.1. Grain Weight, Starch Content, and Sugar Content of BS253 in Response to HT

**Figure 1:**
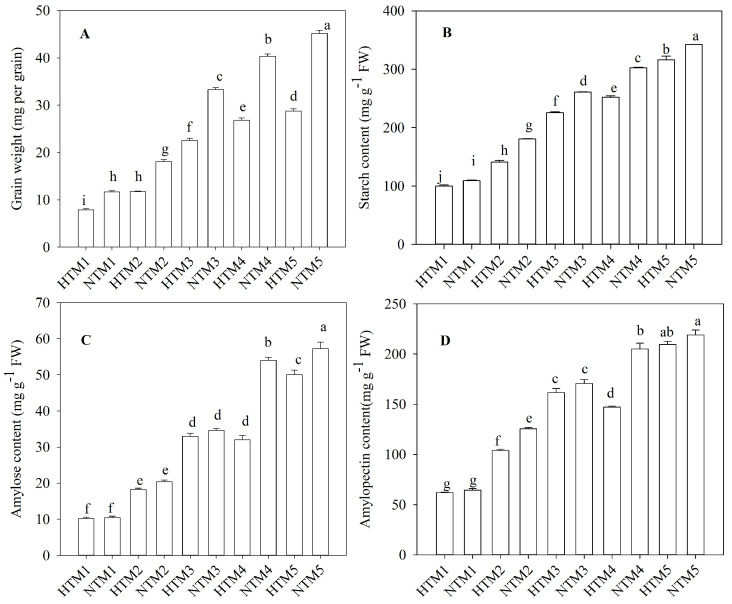
Grain weight (**A**) and starch content (**B**), amylose content (**C**), amylopectin content (**D**) in response to HT in BS253 grains. Bars represent the SE (*n* = 3), and the means with different letters are significantly different at *p* < 0.05.

**Figure 2:**
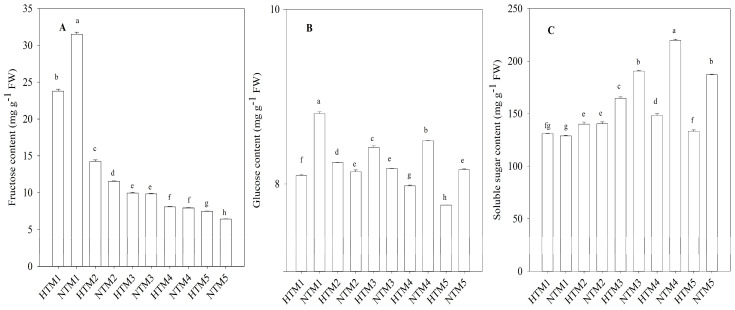
Levels of fructose (**A**), glucose (**B**), and soluble sugars (**C**) in BS253 grains in response to HT. Bars represent the SE (*n* = 3), and the means with different letters are significantly different at *p* < 0.05.

The experiment was divided into five sampling stages: Stage 1 (the first day after heat stress; denoted as HTM1 for heat stress and NTM1 for the control), Stage 2 (the sixth day after heat stress; denoted as HTM2 for heat stress and NTM2 for the control), Stage 3 (the eleventh day after heat stress; denoted as HTM3 for heat stress and NTM3 for the control), Stage 4 (the sixteenth day after heat stress; denoted as HTM4 for heat stress and NTM4 for the control), and Stage 5 (the twenty-first day after heat stress; denoted as HTM5 for heat stress and NTM5 for the control). The weight of the grain and its starch content, which consists of both amylose and amylopectin, gradually increased during the grain filling stage, reaching a maximum at 34 days post-anthesis (DPA; Figure 1). However, HT markedly reduced the starch content, leading to a substantial decrease in grain weight. As grain filling progressed, sucrose content in the grains steadily declined. Notably, our previous studies have indicated that sucrose levels in HTM1 and HTM2 were significantly higher than those in NTM1 and NTM2, respectively. In contrast, sucrose levels in HTM4 and HTM5 were significantly lower than those in NTM4 and NTM5, respectively [31]. The fructose level also decreased throughout the grain-filling process. Fructose levels in NTM1 were substantially higher than those in HTM1, although HT increased fructose content during the later stages of grain filling (Figure 2A). Apart from the significantly higher glucose content in HTM3 than in NTM3, HT also reduced glucose levels in grains (Figure 2B). Furthermore, HT significantly increased the soluble sugar content in HTM1 but markedly reduced it during the later grain-filling stages (Figure 2C).

### 2.2. Enzymatic Activity Involved in Starch Synthesis in Response to HT

**Figure 3:**
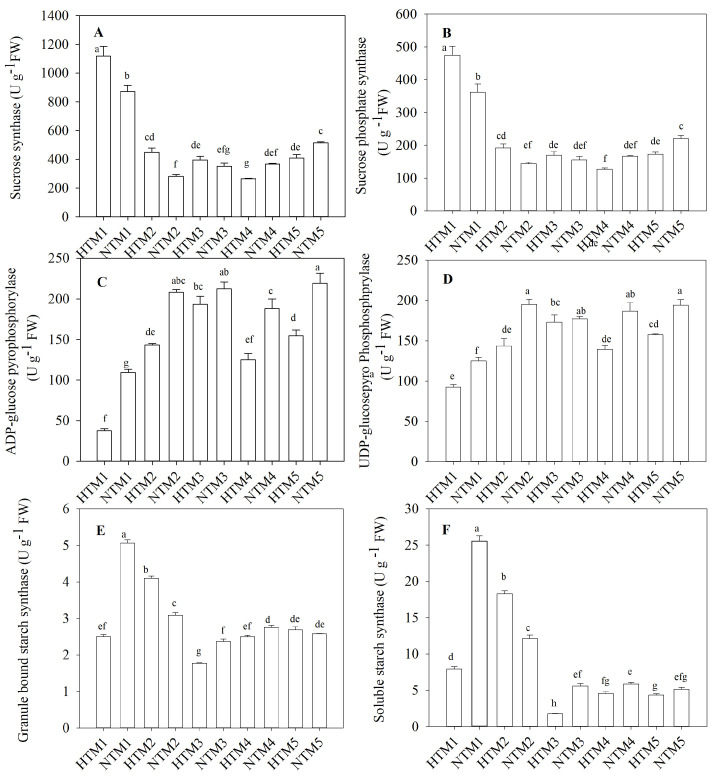
Activity of enzymes involved in starch synthesis in BS253 grains in response to HT (**A**–**F**). Bars represent the SE (*n* = 3), and the means with different letters are significantly different at *p* < 0.05.

The research findings indicated that SuS activity was considerably higher in HTM1 and HTM2 than in NTM1 and NTM2, respectively, whereas HTM4 and HTM5 exhibited markedly lower SuS activity than NTM4 and NTM5 (Figure 3A). No significant differences in SuS activity were observed between HTM3 and NTM3. The changes in SPS activity in response to HT closely followed the pattern observed for SuS activity (Figure 3B). Furthermore, this study revealed that HT significantly reduced AGP activity in grains throughout the grain-filling stage (Figure 3C), and a similar reduction was observed in UGP activity (Figure 3D). The activity of GBSS and SSS also declined in response to HT stress. However, HTM2 exhibited a significant increase in the activity of these enzymes compared with NTM2.

### 2.3. Ultrastructure of Wheat Caryopsis Abdominal Phloem in Response to HT 

**Figure 4:**
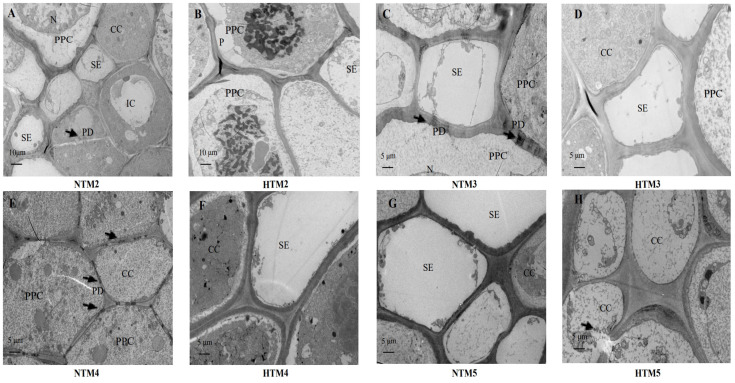
Transmission electron micrographs showing the ultrastructure of abdominal phloem tissue in wheat caryopsis in response to HT. SEs, sieve elements; CCs, companion cells; ICs, intermediary cells; PPC, phloem parenchyma cells; PD, plasmodesmata; and N, nucleus. (**A**,**B**) HT-induced change six days after heat stress treatment; the heat stress treatment and control groups are depicted as HTM2 and NTM2, respectively. (**C**,**D**) Change induced 11 days after heat-stress treatment; the heat-stress treatment and control groups are depicted as HTM3 and NTM3, respectively. (**E**,**F**) Change induced 16 days after heat-stress treatment; the heat-stress treatment and control groups are depicted as HTM4 and NTM4, respectively. (**G**,**H**) Change induced 21 days after heat-stress treatment; the heat-stress treatment and control groups are depicted as HTM5 and NTM5, respectively. The black arrows indicate PD, plasmodesmata.

Figure 4 presents transmission electron micrographs showing the ultrastructure of the phloem in wheat grains at different stages of grain filling. In the majority of SEs (sieve elements), the phloem was associated with CCs (companion cells), and these SEs shared contiguous walls with PPCs (phloem parenchyma cells). Under normal conditions, PPCs at 18 and 23 DPA exhibited a well-defined structure characterized by a prominent nucleus. The PPCs had an irregular shape, with their cell walls invaginating or protruding into adjacent cells, thereby increasing the surface area of the cell membrane and facilitating material transport (Figure 4A,C). Numerous plasmodesmata were observed between SEs and ICs (intermediary cells), as well as between CCs and PPCs (Figure 4A,C,E) under normal conditions. In NTM5, the SEs and CCs were closely aligned and maintained a regular shape, reflecting their role in photo-assimilate transport (Figure 4G). However, under HT conditions, the heterochromatin content in the PPCs of HTM2 increased, and cytoplasmic wall separation occurred (Figure 4B), suggesting a decline in function. In addition, the study found no plasmodesmata between CCs and PPCs or between CCs and other CCs (Figure 4D,F). As grain filling progressed, the gap between SEs and CCs widened in HTM5, and the cell walls of CCs and PPCs began to degrade (Figure 4H).

### 2.4. Overview of Transcriptome Sequencing and Kyoto Encyclopedia of Genes and Genomes Analysis

The RNA-seq analysis generated approximately 323.86 Gb of clean data from 30 samples, with a Q30 percentage exceeding 88.53% (Table S2). Alignment of the clean data with the reference genome resulted in a correspondence rate ranging from 80.6% to 89.42% (Table S3). Samples with a Pearson correlation coefficient (r) below 0.8 were excluded, ensuring that the remaining samples exhibited strong biological reproducibility (Figure S1). Gene expression levels were quantified using fragments per kilobase of transcript sequence per million base pairs sequenced, indicating an effective sampling procedure and high-quality bioinformatics analysis.

Kyoto Encyclopedia of Genes and Genomes (KEGG) pathway analysis was performed to identify the metabolic pathways associated with differentially expressed genes (DEGs). The upregulated DEGs in the HTM1 versus NTM1 comparison showed significant enrichment in the pathways related to glycine, serine, and threonine metabolism, galactose metabolism, and glutathione metabolism. Additionally, DNA replication and mismatch repair pathways were prominently enriched in the HTM2 versus NTM2, HTM3 versus NTM3, and HTM4 versus NTM4 comparisons (Figure S2). By contrast, the analysis of downregulated DEGs in HTM1 versus NTM1 highlighted significant enrichment in pathways associated with photosynthesis-antenna proteins. As grain filling progressed, downregulated DEGs in HTM2 versus NTM2, HTM3 versus NTM3, HTM4 versus NTM4, and HTM5 versus NTM5 were enriched in the pathways related to starch and sucrose metabolism, carbon fixation in photosynthetic organisms, photosynthesis-antenna proteins, and photosynthesis itself (Figure S3).

### 2.5. Gene Ontology Analysis and the Enzyme Activity of Antioxidant System as Well as the Response of Hydrogen Peroxide and Malondialdehyde to HT

**Figure 5:**
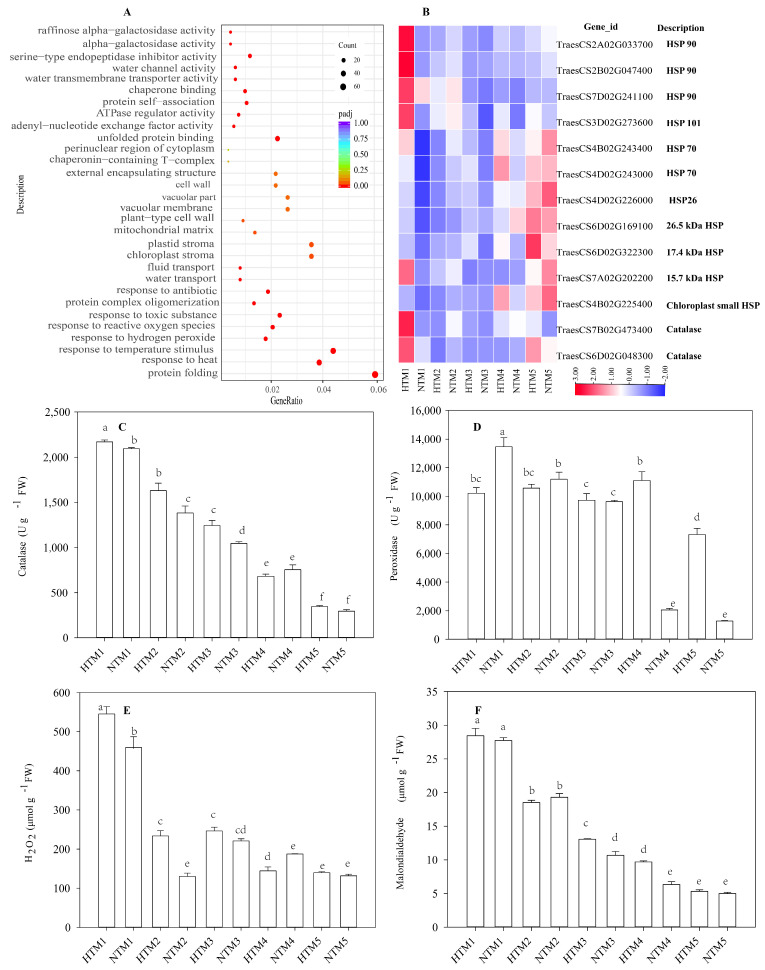
GO analysis and the enzyme activity of the antioxidant system, as well as the response of H_2_O_2_ and MDA to HT. (**A**) GO analysis of upregulated DEGs in HTM1 versus NTM1. (**B**) HSPs and catalase gene expressions in BS253 grains in response to HT. The colors in the heatmap indicate gene expression levels across different samples. (**C**,**D**) Enzyme activity of the antioxidant system in response to HT. Bars represent the SE (*n* = 3). (**E**,**F**) H_2_O_2_ and MDA contents in response to HT. Bars represent the SE (*n* = 3), and the means with different letters are significantly different at *p*  <  0.05.

Gene ontology (GO) analysis of upregulated DEGs revealed significant enrichment in pathways related to responses to hydrogen peroxide (H_2_O_2_), temperature stimuli, heat stress, and protein folding in HTM1 versus NTM1 (Figure 5A). Among these DEGs, we identified 11 HSP genes, including 3 HSP90, 1 HSP101, 2 HSP70, and 5 small HSPs, as well as 2 catalase (CAT) genes (Figure 5B). HT markedly elevated the expression levels of these HSP and CAT genes (*TraesCS7B02G473400* and *TraesCS6D02G048300*) in HTM1 compared with NTM1, suggesting their critical role in HT responses. 

Further studies demonstrated that HT not only increased H2O2 and malondialdehyde (MDA) levels in grains but also significantly enhanced the activities of CAT and peroxidase (POD). In this study, the concentration of H2O2 showed a sharp increase immediately following HT exposure (Figure 5E), which coincided with a concurrent rise in CAT activity (Figure 5C). In contrast, during the later stages following HT exposure, MDA content and POD activity in HTM significantly increased compared with NTM (Figure 5D,F).

### 2.6. HT Response of Key Gene Expression Related to the Pathway from Sucrose Unloading to Starch Synthesis 

**Figure 6:**
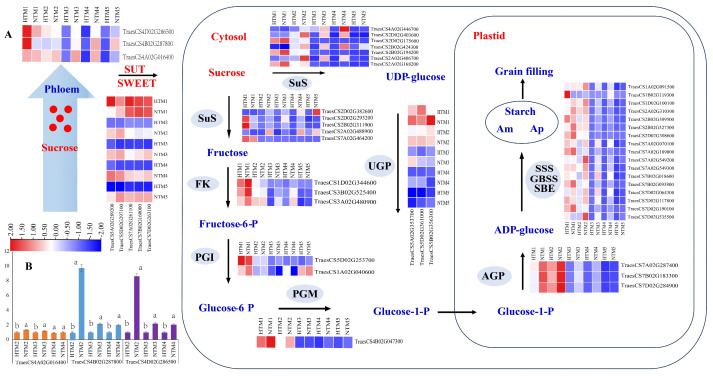
Expression of key genes related to the pathway from sucrose unloading to starch synthesis under HT stress. SuS, sucrose synthases; FK, fructokinase; PGI, phosphoglucoisomerase; PGM, phosphoglucomutase; AGP, ADP-glucose pyrophosphorylase; SSS, soluble starch synthase; GBSS, granule-bound starch synthase; SBE, starch branching enzymes; Am, amylose; Ap, amylopectin. (**A**) Expression patterns of key genes related to the pathway from sucrose unloading to starch synthesis. The colors in the heatmap indicate the expression levels of genes across different samples. (**B**) qRT-PCR was used to verify the expression profile of selected genes (*TraesCS4D02G286500*, *TraesCS4B02G287800*, and *TraesCS4A02G016400*). Bars represent the SE (*n* = 3), and the means with different letters are significantly different at *p* < 0.05.

KEGG pathway analysis indicated that sucrose supply and starch synthesis are potential limiting factors in grain filling under HT conditions. To further elucidate the molecular and physiological mechanisms by which HT regulates grain filling, we examined the expression patterns of genes involved in sucrose unloading and starch synthesis. As shown in Figure 6, we analyzed the expression levels of three *TaSUT* genes (*TraesCS4D02G286500*, *TraesCS4B02G287800*, and *TraesCS4A02G016400*) and five *TaSWEET* genes (*TraesCS7D02G263100*, *TraesCS7B02G160000*, *TraesCS7A02G261100*, *TraesCS5D02G297100*, and *TraesCS5A02G289200*), which are implicated in the processes of sucrose unloading and transport to the grains. The results revealed that although HT initially increased the expression of *SUTs* in the grains, a subsequent decrease in their expression occurred as grain filling progressed.

According to the KEGG analysis, 41 key regulatory genes involved in starch and sucrose metabolic pathways were identified, including 12 *SuS* genes, 3 fructokinase genes, 2 phosphoglucoisomerase genes, 1 phosphoglucomutase gene, 3 UGP genes, 3 AGP genes, and 17 starch synthase-related genes. Gene expression profiling indicated that HT significantly upregulated the expression of *SuS* (*TraesCS2D02G293200* and *TraesCS2B02G311900*) and *GPI* (*TraesCS5D02G253700*) in HTM1 compared with NTM1, corresponding to the observed increase in SuS enzyme activity. However, as grain filling progressed, HT caused a significant reduction in *SuS* expression in the later stages. Similarly, the expression patterns of genes encoding FK, PGI, UGP, PGM, AGP, and those involved in starch synthesis showed a decrease under HT conditions. This reduction in gene expression was more pronounced in comparisons between HTM1 and NTM1 and between HTM2 and NTM2.

### 2.7. Weighted Gene Co-Expression Network Analysis (WGCNA)

**Figure 7:**
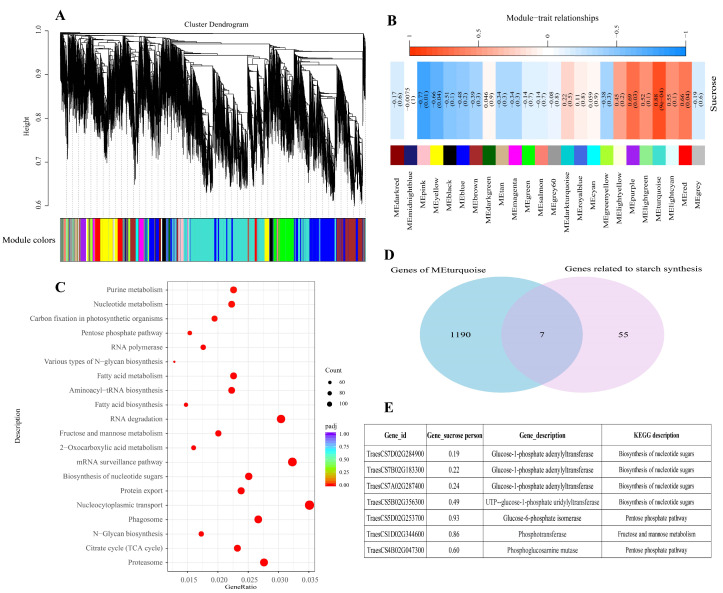
Weighted gene co-expression network analysis. (**A**) A hierarchical clustering tree of genes was constructed based on the co-expression network analysis. (**B**) Module-and-sucrose content correlation. (**C**) KEGG enrichment analysis for genes from turquoise module. (**D**) Venn diagrams between significantly enriched genes (turquoise module) and the identified genes in the pathway from sucrose unloading to starch synthesis. (**E**) Hub genes in the starch synthesis pathway.

According to our results, a total of 24 co-expression modules were identified, and each module was represented by a different color (Figure 7A). The turquoise module was highly positively correlated with the sucrose content (*R* = 0.88, *p* = 0.0009) (Figure 7B). We used the turquoise module genes for KEGG analysis and found that these genes were mainly enriched in the nucleocytoplasmic transport, biosynthesis of nucleotide sugars, and other pathways (Figure 7C). Subsequently, we used 1197 significantly enriched genes and 62 genes, including sucrose-to-starch synthesis and HSPs, for Venn analysis to identify the key genes of the sucrose-to-starch synthesis pathway (Figure 7D). The results showed that we found seven key genes, which mainly involved the pathway of biosynthesis of nucleotide sugars, fructose and mannose metabolism, and the pentose phosphate pathway (Figure 7E). Gene expression analysis showed that HT significantly increased the expression of heat shock protein (*TraesCS3D02G273600*) in grains and significantly decreased the activity of enzymes related to starch synthesis (*TraesCS7D02G284900*, *TraesCS7B02G183300*, and *TraesCS5B02G356300*) (Figure S4).

### 2.8. Analysis of Factors Limiting Grain Filling in BS253

**Figure 8:**
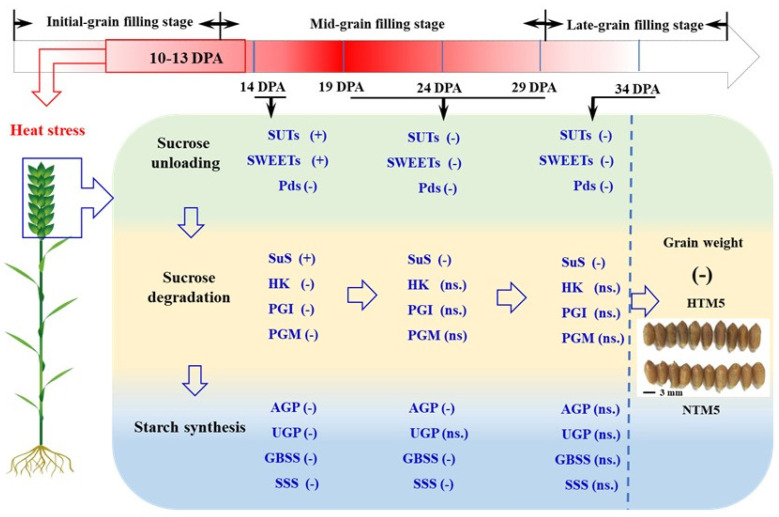
Analysis of factors limiting grain filling in sterile lines. SuS, sucrose synthases; FK, fructokinase; PGI, phosphoglucoisomerase; PGM, phosphoglucomutase; AGP, ADP-glucose pyrophosphorylase; SSS, soluble starch synthase; GBSS, granule-bound starch synthase; SBE, starch branching enzymes; DPA, days post-anthesis; ns., no significant difference; PD, plasmodesmata; (+), HT significantly upregulated the expression levels of relevant genes compared with the control; (−), HT significantly downregulated the expression levels of relevant genes compared with the control. The twenty-first day after heat stress treatment is represented as HTM5, and NTM5 was the control.

This study explored the molecular and physiological mechanisms underlying grain filling under HT conditions, focusing on three key processes, namely sucrose unloading and transport, sucrose degradation, and starch synthesis across different stages of grain filling. As illustrated in Figure 8, during the early phase following HT exposure, the expression of sucrose transporter proteins and genes involved in sucrose degradation, such as *TaSUT1* and *TaSWEET*, increased significantly. This finding correlated with the observed increase in the sucrose content during this period. However, in the later stages, HT substantially reduced the expression of key genes involved in starch synthesis, including *PGM*, *PGI*, *AGP*, *GBSS*, and *SSS*. By integrating the structural characteristics of SE-CC complexes in developing grains, we constructed a schematic model that illustrates the impact of HT on grain filling. The initial limitation of grain filling under HT is primarily due to the disruption of starch synthesis. As grain filling progresses, both the restricted sucrose supply and the impaired starch synthesis become the main limiting factors for successful grain development under HT conditions.

## 3. Discussion

### 3.1. Pathways from Sucrose Unloading to Starch Synthesis in Response to HT

In regions where hybrid wheat seeds are produced, HT is a common abiotic stress during the grain-filling stage. Statistics indicate that the number of days with maximum temperatures exceeding 30 °C during grain filling can reach up to 20 days, which has a detrimental impact on this critical phase. Because both sucrose unloading and subsequent transport in outcrossing and self-pollinated grains occur within maternal tissues [7], studying the response of these processes, along with starch synthesis, to HT in the reproductive zone of PTMS lines provides a foundation for understanding outcrossing grain filling. In line with previous studies on crops [32,33], HT markedly reduced starch content and grain weight in PTMS lines (Figure 1). Sucrose is converted into starch in grains through three main stages: sucrose unloading and transport, sucrose degradation, and starch synthesis in sink tissues [34]. Sucrose unloading occurs along the entire SE-CC complex in the dorsal vascular bundles of grains. HT disrupts cellular and subcellular structures, adversely affecting crop yield [35]. Similar to the responses of the SE-CC complex to drought [34], HT caused the detachment of heterochromatin from the cytoplasmic walls in PPC cells and widened the intercellular spaces between adjacent pericytes in the SE-CC complex, thereby impairing grain filling. Previous studies have implicated cereal SUTs and SWEETs in phloem unloading [22,36], with evidence suggesting that this process involves apoplastic unloading in grain tissues [37,38]. To date, seven *TaSUT* and *TaSWEET* genes have been identified [20,31,39]. Consistent with findings in rice and *Arabidopsis*, where heat stress downregulates *SUT* expression [33], our results demonstrated that HT initially upregulated *TaSUT1* (*TraesCS4D02G286500*, *TraesCS4B02G287800*, and *TraesCS4A02G016400*), followed by a subsequent decrease in expression in HTM1 compared with NTM1. Similarly, *TaSWEET* genes (*TraesCS7D02G263100*, *TraesCS7B02G160000*, *TraesCS7A02G261100*, *TraesCS5D02G297100*, and *TraesCS5A02G289200*) displayed the same trend in response to HT as *TaSUT1*. In addition, the trends in sucrose and soluble sugar contents in grains following HT mirrored the expression patterns of SUTs, indicating that sucrose supply is not a limiting factor during the initial phase post-HT. To further investigate the roles of *TaSUT1*, we developed CRISPR/Cas9-based gene knockout and overexpression vectors, as previously described.

The region between the nuchal protrusions on the inner surface of the SE-CC complex and the endosperm cells is commonly referred to as the nutrient pool (or endosperm cavity). In this cavity, sucrose is initially unloaded through both symplastic and apoplastic pathways. From there, sucrose is transported into the seed’s endosperm through aleurone layer cells or endosperm transfer cells due to the symplastic isolation between the endosperm cavity and the endosperm cells in wheat grains [30,38,40]. Although the roles of SUT and SWEET proteins in sugar unloading have been well-established in other crops [41,42,43], such as *SUT* genes facilitating sucrose translocation between the apoplast and phloem and SWEET genes being localized in phloem parenchyma [22,44], the specific roles of SUTs in unloading or in subsequent transport to the endosperm in PTMS grains remain unclear. Future studies should use advanced techniques, such as immunohistochemistry and in situ hybridization, to clarify the functions of the SUTs identified in this study.

The comprehensive pathway from sucrose unloading to starch synthesis has been extensively detailed (Figure 6) based on the existing literature [45]. The process begins with the cleavage of sucrose in the cytosol, catalyzed by SuS, which converts sucrose into UDP-glucose and fructose [46]. Three haplotypes, namely *TaSuSy1*, *TaSuSy2*, and *TaSuSy3*, have been linked to variations in grain weight [19,47]. Previous research has demonstrated that heat and drought stress can affect the activity of enzymes involved in sucrose-to-starch metabolism [48], generally leading to reduced expression of genes such as *TaAGP*, *TaGBSS1*, *TaSBEI*, and *TaSBEII* [25]. Further investigations have identified 41 key regulatory genes in the sucrose-to-starch synthesis pathway through KEGG analysis. Our experimental findings indicated that *SuS* genes (*TraesCS2D02G293200* and *TraesCS2B02G311900*) were significantly upregulated during the initial phase post-HT. In contrast, genes involved in starch synthesis, including *TaAGP*, *TaGBSS*, *TaSSS*, and *TaSBE*, were consistently downregulated post-HT.

Correspondingly, the enzymatic activities crucial for starch synthesis also declined markedly under HT conditions (Figure 3). These data suggest that the reduction in gene expression within the starch synthesis pathway limits grain filling during the early stages post-HT (Figure 7). We also identified seven key genes related to the biosynthesis of nucleotide sugars, fructose and mannose metabolism, and the pentose phosphate pathway by *WGCAN*, and our next step is to conduct research on the functions of these genes. Based on gene expression patterns related to sucrose-to-starch synthesis during the mid-to-late grain-filling stages post-HT, we conclude that the inhibition of both sucrose transport and starch synthesis becomes a major limiting factor for grain filling. KEGG analysis of downregulated DEGs revealed significant enrichment in metabolic pathways related to carbon fixation, photosynthesis-antenna proteins, and photosynthesis throughout the grain-filling stage post-HT. This finding suggests that insufficient production of photosynthetic products may be a critical factor limiting grain filling. Previous studies have shown that photosynthesis in flag leaves and spikes contributes 12% and 20.1% of photosynthetic substances, respectively [49]. However, the contribution of grain photosynthesis to overall yield has not yet been reported. Thus, the findings of this study offer a novel perspective for future research into the role of grain photosynthesis in yield formation.

### 3.2. HSPs and Antioxidant System in Response to HT

HT affects various metabolic pathways [35] in crops, which can exhibit thermotolerance through specific heat-resistance mechanisms [14]. Currently, 753 HSP genes have been identified in the wheat genome, including 169 *TaSHSP*, 273 *TaHSP40*, 95 *TaHSP60*, 114 *TaHSP70*, 18 *TaHSP90*, and 84 *TaHSP100* [50]. The upregulation of these genes plays a crucial role in wheat’s response to HT [14,51]. Wheat chloroplastic small HSPs, such as HSP26 and HSP17.4, have been shown to enhance tolerance to heat stress [52,53]. HSP100/ClpB collaborates with its cognate HSP70 partner to refold toxic protein aggregates back into their native state [54]. However, the biological roles of *TaSHSPs* in regulating grain filling under HT conditions remain poorly understood. In this study, we observed significant enrichment in pathways related to hydrogen peroxide response, temperature stimuli, heat response, and protein folding in HTM1 versus NTM1. We identified 11 *HSP* genes, including 3 *HSP90*, 1 *HSP101*, 2 *HSP70*, and 5 small HSPs (*HSP26.5*, *HSP26*, *HSP17.4*, *HSP15.7*, and chloroplastic small HSP) with the expression of *HSP101*, *HSP90*, and *HSP15.7* significantly upregulated compared to other HSPs. Our findings suggest that the expression of some *TaHSP* genes mirrors the trends observed for *TaSUT1* and *TaSuS* in the initial phase post-HT. Previous studies have shown that *HSP101* in rice directly interacts with key enzymes involved in starch synthesis, such as *AGPL1*, *AGPL3*, and *PHO1* [55]. Additionally, *TaHSP17.4* has been reported to associate with the HSP70/HSP90 organizing protein (HOP) in wheat in response to HT, suggesting a universal adaptive mechanism to cope with environmental stress [53]. Collectively, these findings imply that the upregulation of *TaHSPs* may regulate sucrose unloading, sucrose degradation, and the activities of AGP and UGP, thereby influencing grain filling. However, the precise molecular mechanisms by which *TaHSPs* regulate these processes under HT conditions in wheat require further investigation.

H_2_O_2_, a reactive oxygen species (ROS), can be significantly induced by HT, and antioxidant defense systems, including catalase (CAT) and peroxidase (POD), play crucial roles in scavenging ROS in crops [56]. This study shows that the trends in H_2_O_2_ content and CAT activity were consistent during the initial phase post-HT. Furthermore, the expression of CAT genes was significantly upregulated during this period, suggesting that CAT plays a critical role in removing H_2_O_2_ and maintaining a favorable cellular microenvironment following HT stress, consistent with findings in cucumber [35]. Interestingly, we also observed that the expression levels of *TaSHSP* genes and CAT activity exhibited similar trends in response to HT. Previous studies have shown that the transcription levels of CAT and POD were significantly higher in *TaHSP17.4*-overexpressing plants compared to wild-type plants under stress conditions [53], suggesting that HSPs help maintain a favorable cellular environment by regulating antioxidant enzyme activities in PTMS grains under HT conditions. However, further molecular-level research is required to elucidate how HSPs contribute to the regulation of the grain-filling process in PTMS by modulating the antioxidant defense system under HT stress.

## 4. Materials and Methods

### 4.1. Plant Materials, Experimental Design, and Sampling

The experiments were conducted during the 2021–2022 growing seasons at the Experimental Station of the Beijing Hybrid Wheat Research Institute in Haidian, Beijing, China (39°56′ N, 116°17′ E). PTMS lines, which are crucial for the two-line hybrid wheat breeding system, exhibit high sterility in hybrid seed production areas (approximately 32° N latitude) and restored fertility in reproductive areas (approximately 40° N latitude). This experiment was conducted in the reproductive area. The BS253 line was planted in six plots, with three plots designated for HT stress treatment and the remaining three serving as controls. The experimental site had loam soil. Throughout three growing seasons, 120 kg N ha^−1^, 75 kg P_2_O_5_ ha^−1^, and 120 kg K_2_O ha^−1^ were applied as basal fertilizer before planting, with an additional 120 kg N ha^−1^ being top-dressed at the jointing stage. Each plot measured 3 m × 1 m with a row spacing of 0.25 m and a planting density of 100 plants m^−2^. The HT stress treatment involved constructing a 1.5-m-high shed from hollow pipes and covering it with a 0.1 mm-thick white polythene film with 90% light transmittance. This structure was set up with 10 DPA to elevate temperatures. Four ventilation openings (10 cm × 12 cm) were placed at the top of both sides of the shed to maintain humidity levels similar to that in control plots. HT stress was applied from 8:00 to 18:00 on 15 May and continued for four days, after which the shed was dismantled. Two temperature sensors were placed 0.3 m above the canopy in both the control and HT stress treatment plots. Temperature data were recorded automatically every 5 min during the treatment period. The mean daily temperatures from 8:00 to 18:00 were 35.09 °C and 27.79 °C in the treatment and control plots, respectively.

Wheat anthesis and maturity occurred on 6 May and 12 June 2022, respectively. Because of the split glumes of male-sterile lines during the flowering stage, approximately 200 spikes that flowered on the same day were selected at the heading stage (GS55) and covered with 20 white bags (12 cm × 20 cm) to prevent pollination (10 spikes per bag). For each stage, 40 labeled spikes from both the heat stress treatment and control groups were sampled, with three replicates per stage. Grains from 10 labeled spikes per treatment were used for RNA-seq analysis and gene expression studies, whereas grains from another 10 labeled spikes were used to measure sugar content and enzyme activity related to the sucrose-to-starch pathway. In addition, 20 labeled spikes from each plot at all five stages were sampled and used for grain weight analysis.

### 4.2. Sugar and Starch Content Determination 

The contents of amylopectin and amylose were quantified using the double wavelength method [57], and total starch content was calculated as the sum of these two components. The concentrations of fructose, glucose, and sucrose in the grains were measured using anthrone–sulfuric acid colorimetry [58]. Total soluble sugar content was determined by employing the anthrone method [59], and sucrose was used as the standard.

### 4.3. Enzymatic Activities in the Sucrose-to-Starch Pathway

We initially quantified the activities of SS and SPS by using assay kits specific to these enzymes. We then measured the activities of SSS, GBSS, AGP, and UGP by using corresponding assay kits. All assay procedures were conducted according to the manufacturer’s instructions provided by Nanjing Mofan Biotechnology Co., Ltd. (Nanjing, China). 

### 4.4. Measurement of MDA, CAT, POD, and H_2_O_2_

The determination of CAT, POD, MDA, and H_2_O contents was outsourced to Nanjing Convinced-test Technology Co., Ltd. (Nanjing, China), which provided the necessary technical support. All measurements were performed in triplicate using three biological replicates.

### 4.5. Transmission Electron Microscopy

The ultrastructural features of wheat grain abdominal phloem tissues at 14, 19, 24, 29, and 34 DPA were examined using transmission electron microscopy (TEM) to assess the impact of HT on SE-CC complexes. For TEM preparation, small fragments of vascular tissue were promptly fixed in 2.5% glutaraldehyde (*v*/*v*) at 4 °C for 24 h, followed by five 20-min rinses in 0.1 M phosphate buffer (pH 7.2). The samples were then post-fixed in 1% osmium tetroxide in the same buffer for 5 h. After five additional 20-min washes in phosphate buffer (pH 7.2), the samples were dehydrated through a graded ethanol series [45%, 55%, 70%, 85%, 95%, and 100% (*v*/*v* in distilled water)], with each concentration applied for 30 min at room temperature. Following dehydration, the samples were infiltrated with propylene oxide for 5 h, then immersed in a 1:1 (*v*/*v*) mixture of propylene oxide and resin for 8 h, followed by immersion in 100% (*v*/*v*) resin for another 8 h. The samples were embedded in resin with DMP-30 and polymerized at 60 °C for 24 h. Thin sections (80 to 90 nm) were cut using a Leica EM UC7 ultramicrotome, stained with lead citrate for 30 min, rinsed three times for 1 min in distilled water, and subsequently stained with 2% (*w*/*v*) uranyl acetate in distilled water for 30 min, followed by three more 1-min rinses in distilled water. The transverse sections of phloem tissues were then examined using a Hitachi HT7700 TEM (Tokyo, Japan).

### 4.6. RNA Extraction, cDNA Library Construction, and Transcriptome Sequencing

RNA was extracted using the RNAprep Pure Plant Kit (TIANGEN, Beijing, China). Sequencing libraries were prepared with the NEBNext Ultra RNA Library Prep Kit for Illumina (San Diego, CA, USA), and index codes were added to assign sequences to each sample. The quality of the libraries was assessed using the Agilent Bioanalyzer 2100 system. Clustering of the index-coded samples was performed on a cBot Cluster Generation System using the TruSeq PE Cluster Kit v3-cBot-HS (Illumina). Following cluster generation, the libraries were sequenced on an Illumina Novaseq platform, producing 150 bp paired-end reads.

### 4.7. Transcriptome Data Processing 

Raw data in fastq format were initially processed using custom Perl scripts to generate clean reads, which were then mapped to the IWGSC CS RefSeq v2.1 reference genome assembly. Differential expression analysis was conducted across various sample groups. *p*-values were adjusted using the Benjamini and Hochberg method to control the false discovery rate. Genes with |log_2_FoldChange| ≥ 1 and *padj* < 0.05 were considered differentially expressed. GO enrichment analysis of DEGs was performed using the *clusterProfiler* R package, with correction for gene length bias. GO terms with a corrected *p*-value of <0.05 were deemed significantly enriched. Additionally, KEGG pathway enrichment of DEGs was assessed using the *clusterProfiler* R package.

### 4.8. WGCNA

The WGCNA was constructed using the R package (Version 3.5.0) for the leaf RNA-Seq data. To ensure the distribution of scale-free networks, the weighting coefficient β should meet the correlation coefficient close to 0.8 and have a certain degree of gene connectivity. In this study, β = 9 was selected as the weighting coefficient. Genes with similar expression patterns were categorized into different modules using a bottom-up algorithm with a module minimum size cutoff of 100. The correlation between module eigengenes and the sucrose content was calculated using a Pearson test; *R* > 0.8 and *p* < 0.05 were used as criteria for screening the specificity modules. In addition, turquoise module genes were used for KEGG enrichment analysis, and the significantly enriched genes were correlated with the identified genes in the pathway from sucrose unloading to starch synthesis, and the hub genes in the starch synthesis pathway were listed.

### 4.9. Gene Expression Analysis 

To validate the transcriptome sequencing data and explore the gene expression levels in response to HT, genes such as sucrose transporters (*TraesCS4D02G286500*, *TraesCS4B02G287800*, and *TraesCS4A02G016400*), heat shock protein 101 (*TraesCS3D02G273600*), ADP-glucose pyrophosphorylase (*TraesCS7D02G284900*, *TraesCS7B02G183300*), and UTP--glucose-1-phosphate uridylyltransferase (*TraesCS5B02G356300*) were subjected to quantitative real-time PCR (qRT-PCR) with three biological replicates. The primers (Table S1) used were designed using Primer 5.0. Relative gene expression levels were calculated using the 2^−ΔΔCt^ method, with *Actin* serving as the endogenous control for normalization.

### 4.10. Statistical Analysis

Multivariate analysis of variance was conducted using Data Processing System software (Version 7.05, China), and significant differences were determined using the least significant difference test at *p* < 0.05.

## 5. Conclusions

This study offers a comprehensive understanding of how HT inhibits grain filling, covering the entire process from sucrose unloading to starch synthesis. Specifically, our results suggest that impaired starch synthesis is the primary factor limiting grain filling immediately after HT exposure. As grain filling progresses, limited sucrose supply, sucrose degradation, and further inhibition of starch synthesis collectively become significant factors constraining grain development during the mid and later stages post-HT. Moreover, this research has identified seven key genes associated with sucrose unloading, starch synthesis, and HT stress responses, laying the groundwork for future investigations into their functional roles. The insights gained from this study may inform the development of strategies to mitigate the impact of HT on grain filling, especially as rising temperatures become more prevalent.

## Data Availability

The original contributions presented in the study are included in the article/Supplementary Material. Further inquiries may be directed to the corresponding author.

## Supplementary Materials

The supporting information can be downloaded at: https://www.mdpi.com/article/10.3390/ijms252212230/s1.
