# Supplementary material for: Physiological and Transcriptome Analyses Provide Insights into the Response of Grain Filling to High Temperature in Male-Sterile Wheat (Triticum aestivum L.) Lines
Source: Int J Mol Sci. 2024 Nov 14;25(22):12230. doi: 10.3390/ijms252212230 (PMC11594281; doi:10.3390/ijms252212230)
Supplement: Supplementary file 1 [file ijms-25-12230-s001.zip › Supplementary Figures.pdf]

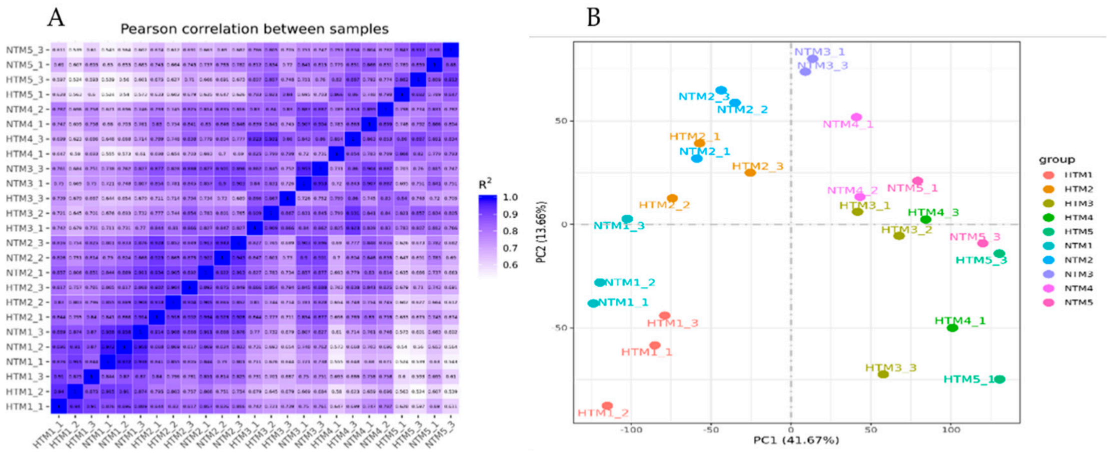

Figure S1. Correlation Heatmap between samples and a principal component analysis (PCA). The horizontal and vertical axes represent the squares of the correlation coefficients between each pair of samples. On the first day following heat stress treatment, the heat-stressed and control groups are designated as HTM1 and NTM1, respectively. The sixth day after heat stress treatment, the heat stress treatment and the control are expressed as HTM2 and NTM2, respectively. The eleventh day after heat stress treatment, the heat stress treatment and the control are expressed as HTM3 and NTM3, respectively. The sixteenth day after heat stress treatment, the heat stress treatment and the control are expressed as HTM4 and NTM4, respectively. The twenty-first day after heat stress treatment, the heat stress treatment and the control are expressed as HTM5 and NTM5, respectively.

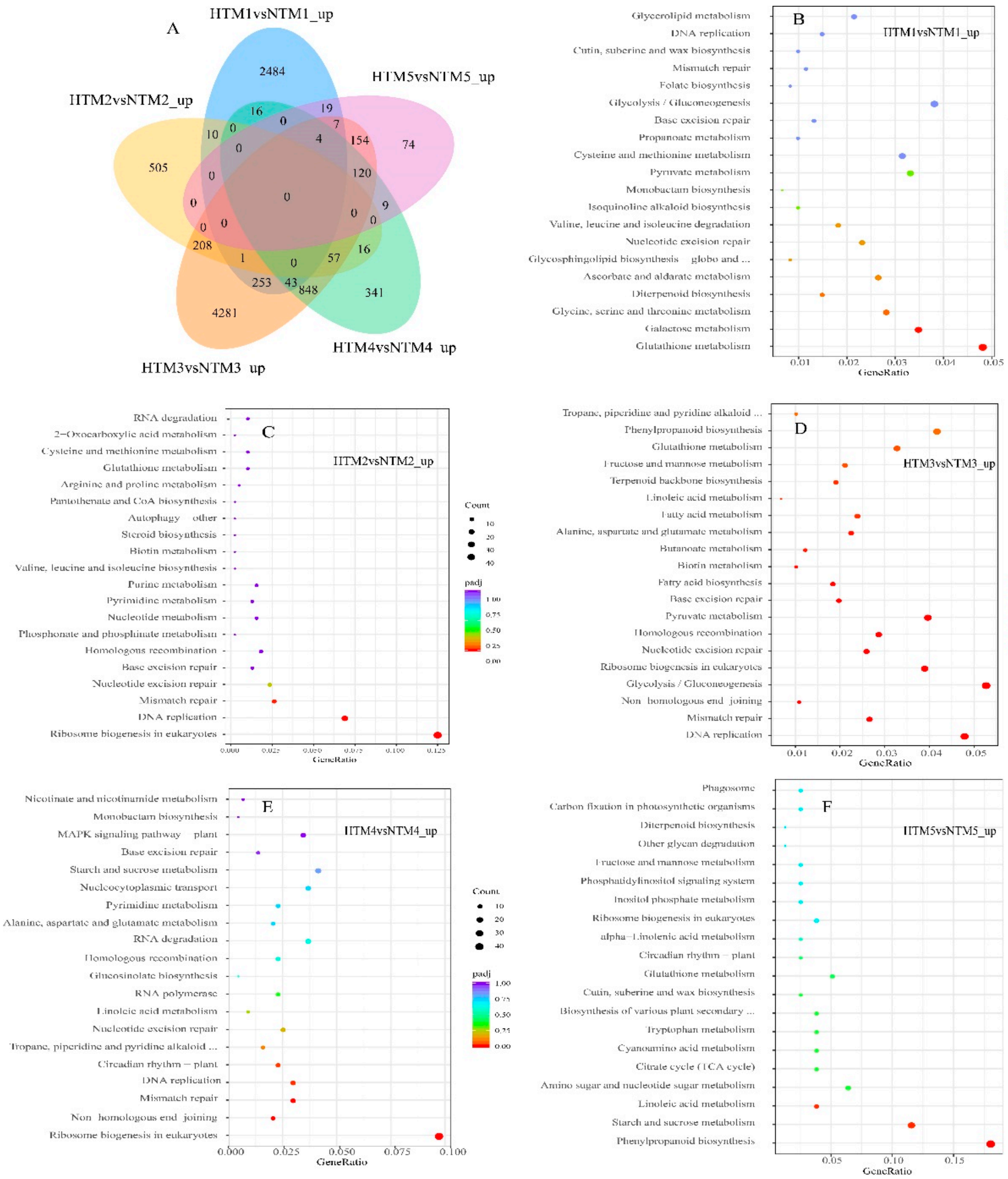

Figure S2. Identification and functional characterization of the up-regulate genes in BS253. (**A**), Venn diagrams showing DEGs between samples from HT and the control at five sampling stages. (**B**,**C**,**D**,**E**,**F**) Kyoto Encyclopedia of Genes and Genomes (KEGG) enrichment analysis of the DEGs in the comparisons of HTM1 vs. NTM1, HTM2 vs. NTM2, HTM3 vs. NTM3, HTM4 vs. NTM4 and HTM3 vs. NTM3. The first day after heat stress treatment, the heat stress treatment and the control are expressed as HTM1 and NTM1, respectively. The sixth day after heat stress treatment, the heat stress treatment and the control are expressed as HTM2 and NTM2, respectively. The eleventh day after heat stress treatment, the heat stress treatment and the control are expressed as HTM3 and NTM3, respectively. The sixteenth day after heat stress treatment, the heat stress treatment and the control are expressed as HTM4 and NTM4, respectively. The twenty-first day after heat stress treatment, the heat stress treatment and the control are expressed as HTM5 and NTM5, respectively.

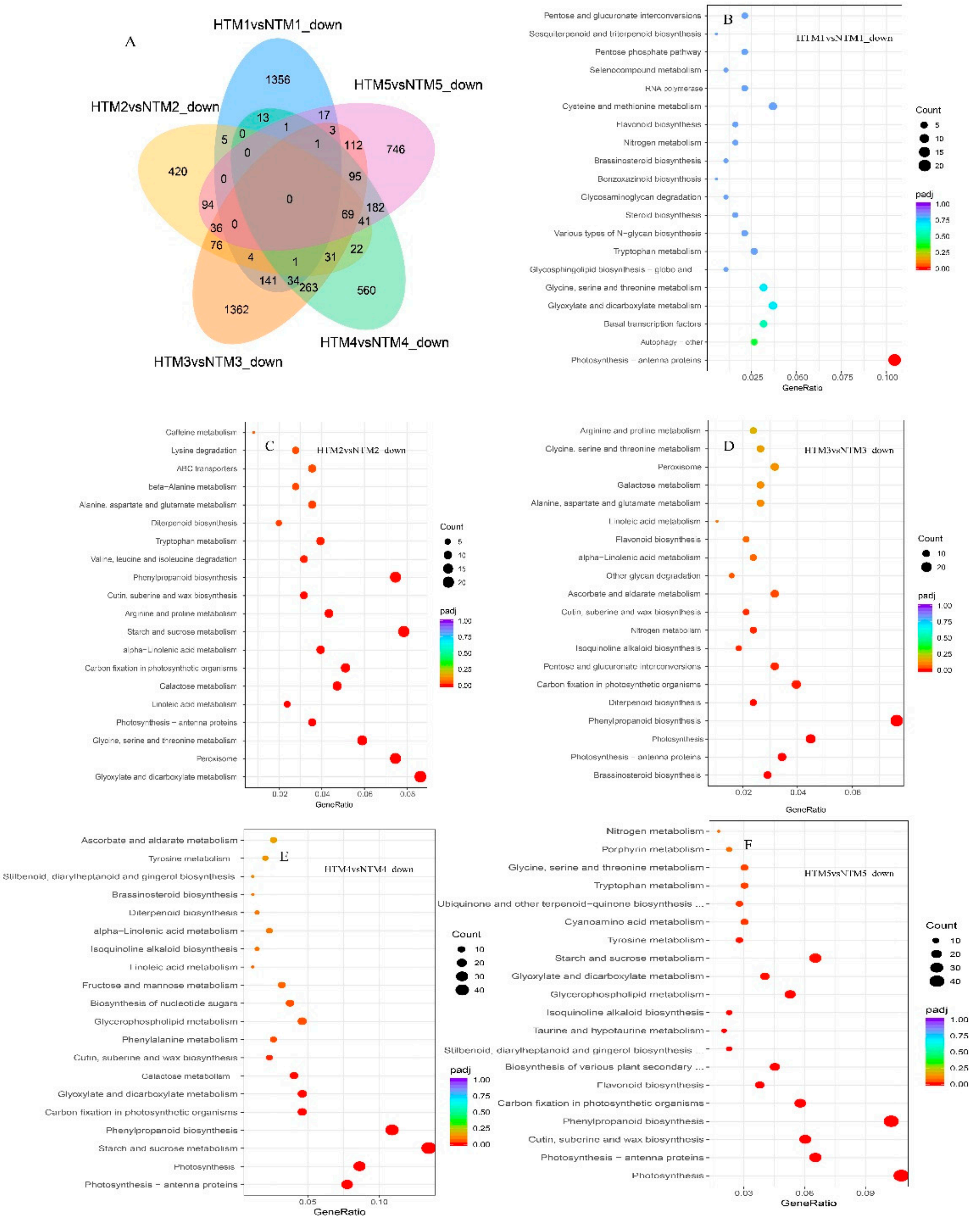

Figure S3. Identification and functional characterization of the down-regulate genes in BS253. (**A**) Venn diagrams showing DEGs between samples from HT and the control at five sampling stages. (**B**,**C**,**D**,**E**,**F**) Kyoto Encyclopedia of Genes and Genomes (KEGG) enrichment analysis of the DEGs in the comparisons of HTM1 vs. NTM1, HTM2 vs. NTM2, HTM3 vs. NTM3, HTM4 vs. NTM4 and HTM3 vs. NTM3. The first day after heat stress treatment, the heat stress treatment and the control are expressed as HTM1 and NTM1, respectively.The sixth day after heat stress treatment, the heat stress treatment and the control are expressed as HTM2 and NTM2, respectively; The eleventh day after heat stress treatment, the heat stress treatment and

the control are expressed as HTM3 and NTM3, respectively. The sixteenth day after heat stress treatment, the heat stress treatment and the control are expressed as HTM4 and NTM4, respectively. The twenty-first day after heat stress treatment, the heat stress treatment and the control are expressed as HTM5 and NTM5, respectively.

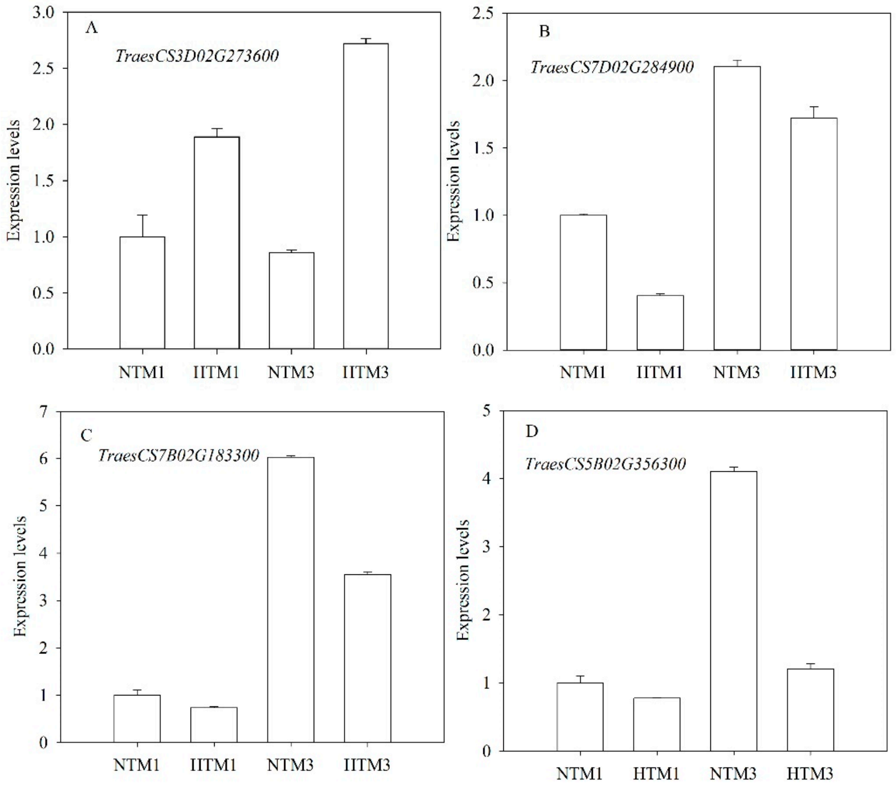

Figure S4. qRT-PCR was used to verify the expression profile of selected genes (*TraesCS3D02G273600*, *TraesCS7D02G284900*, *TraesCS7B02G183300* and *TraesCS5B02G356300*). Bars represent the SE (n = 3). The first day after heat stress treatment, the heat stress treatment and the control are expressed as HTM1 and NTM1, respectively. The eleventh day after heat stress treatment, the heat stress treatment and the control are expressed as HTM3 and NTM3, respectively.
