# Supplementary material for: Physiological and Transcriptome Analyses Provide Insights into the Response of Grain Filling to High Temperature in Male-Sterile Wheat (Triticum aestivum L.) Lines
Source: Int J Mol Sci. 2024 Nov 14;25(22):12230. doi: 10.3390/ijms252212230 (PMC11594281; doi:10.3390/ijms252212230)
Supplement: Supplementary file 1 [file ijms-25-12230-s001.zip › Supplementary Tables.pdf]

Table S1 The primers utilized for qRT-PCR analysis

Gene	Forward primer (5′→3′)	Reverse primer (5′→3′)
TraesCS4B02G287800	TCTCCTCACTCCACGCTCCT	CCTCGTTCGTTCGCCTATC
TraesCS4D02G286500	TCACTCCACGCTCCTTCC	CGTCCGTTCGCCTATCAA
TraesCS4A02G016400	AGGACTCCGTAGAGCAGAATAA	TCACAACCCAAAGACGACAC
TraesCS3D02G273600	ACGCCGTGAAGAAGATG	ATACGATTCCGAACTACCTAA
TraesCS7D02G284900	CGGATGTGGTGGAGATTA	AACGGTTTGTGAGGAAGAG
TraesCS7B02G183300	TATGAGCAATAGCCAGTC	ACCCAGGTTCAAGTCC
TraesCS5B02G356300	GCGAGAACGAGAAGTCCG	AACCACCCGTCAGCAAGT

Table S2 Overview of the transcriptome sequencing(Clean data)

Samples	Raw Reads	Clean Reads	Clean Base (Gb)	Error Rate (%)	Q20 (%)	Q30 (%)	GC Content (%)
HTM1_1	77792420	73571698	11.04	0.03	95.51	89.27	53.63
HTM1_2	72652816	68638872	10.3	0.03	95.92	89.95	54.18
HTM1_3	73967694	71312596	10.7	0.03	95.49	89.5	52.7
NTM1_1	74507996	71926966	10.79	0.03	95.64	89.57	53.99
NTM1_2	74594802	71865186	10.78	0.03	95.56	89.5	53.72

NTM1_3	72976086	69845888	10.48	0.03	95.01	88.53	52.83
HTM2_1	74315850	72453616	10.87	0.03	95.24	88.96	50.42
HTM2_2	76298052	73794494	11.07	0.03	95.84	89.96	50.79
HTM2_3	74530708	70438496	10.57	0.03	95.4	89.31	52.74
NTM2_1	73706626	71541924	10.73	0.03	95.29	88.96	51.49
NTM2_2	74292818	72180262	10.83	0.03	95.76	89.75	50.83
NTM2_3	76624360	74655254	11.2	0.03	95.3	89.01	50.54
HTM3_1	67763090	66009568	9.9	0.03	95.18	88.8	50.61
HTM3_2	76012820	73735838	11.06	0.03	95.93	90.62	53.56
HTM3_3	71021910	69629756	10.44	0.03	96.63	91.89	54.72
NTM3_1	73312298	71550762	10.73	0.03	95.31	89.46	51.28
NTM3_2	73127570	71748010	10.76	0.03	96.22	91.06	54.41
NTM3_3	73587156	71563032	10.73	0.03	95.91	90.64	51.25
HTM4_1	75829652	72875350	10.93	0.03	95.21	88.99	50.14
HTM4_2	72505420	70733800	10.61	0.03	95.4	89.69	51.06
HTM4_3	79086312	77221690	11.58	0.03	95.65	90.11	51.57
NTM4_1	72981488	70697556	10.6	0.03	95.83	90.35	51.13
NTM4_2	77140342	74513376	11.18	0.03	96.26	91.08	52.29

NTM4_3	76674088	74686518	11.2	0.03	96.75	91.99	55.19
HTM5_1	77768344	76301712	11.45	0.03	96.76	91.97	54.8
HTM5_2	72748076	71241782	10.69	0.03	96.33	91.31	55.1
HTM5_3	73583842	72130156	10.82	0.03	96.27	91.08	55.1
NTM5_1	70801154	69427786	10.41	0.03	96.17	90.92	53.11
NTM5_2	72857376	70824832	10.62	0.03	95.31	89.73	53.15
NTM5_3	72961296	71909312	10.79	0.03	96.95	92.12	56.02

Table S3 Overview of the transcriptome sequencing(Compared to reference genome)

Samples	Total Reads	Reads mapped	Unique mapped	Multi mapped	Read1 mapped	Read2 mapped	'+' mapped	'-' mapped
HTM1_1	73571698	65480335(89.0%)	61445011(83.52%)	4035324(5.48%)	31042715(42.19%)	30402296(41.32%)	30659788(41.67%)	30785223(41.84%)
HTM1_2	68638872	62860450(91.58%)	59426965(86.58%)	3433485(5.0%)	29915273(43.58%)	29511692(43.0%)	29656367(43.21%)	29770598(43.37%)
HTM1_3	71312596	62620975(87.81%)	58302709(81.76%)	4318266(6.06%)	29340261(41.14%)	28962448(40.61%)	29101793(40.81%)	29200916(40.95%)
NTM1_1	71926966	62641759(87.09%)	59400259(82.58%)	3241500(4.51%)	29903353(41.57%)	29496906(41.01%)	29661017(41.24%)	29739242(41.35%)
NTM1_2	71865186	61723049(85.89%)	58088109(80.83%)	3634940(5.06%)	29230707(40.67%)	28857402(40.15%)	28998117(40.35%)	29089992(40.48%)

NTM1_3	69845888	57030416(81.65%)	53399807(76.45%)	3630609(5.2%)	26991611(38.64%)	26408196(37.81%)	26667939(38.18%)	26731868(38.27%)
HTM2_1	72453616	59018492(81.46%)	54220438(74.83%)	4798054(6.62%)	27381904(37.79%)	26838534(37.04%)	27079719(37.38%)	27140719(37.46%)
HTM2_2	73794494	61590118(83.46%)	57325943(77.68%)	4264175(5.78%)	28859986(39.11%)	28465957(38.57%)	28668715(38.85%)	28657228(38.83%)
HTM2_3	70438496	57438765(81.54%)	52904880(75.11%)	4533885(6.44%)	26623008(37.8%)	26281872(37.31%)	26433618(37.53%)	26471262(37.58%)
NTM2_1	71541924	58335337(81.54%)	52930203(73.98%)	5405134(7.56%)	26716654(37.34%)	26213549(36.64%)	26420606(36.93%)	26509597(37.05%)
NTM2_2	72180262	60631766(84.0%)	55284868(76.59%)	5346898(7.41%)	27846457(38.58%)	27438411(38.01%)	27633009(38.28%)	27651859(38.31%)
NTM2_3	74655254	61090879(81.83%)	55598360(74.47%)	5492519(7.36%)	28078800(37.61%)	27519560(36.86%)	27742062(37.16%)	27856298(37.31%)
HTM3_1	66009568	53203471(80.6%)	47932856(72.62%)	5270615(7.98%)	24168819(36.61%)	23764037(36.0%)	23997453(36.35%)	23935403(36.26%)
HTM3_2	73735838	57751725(78.32%)	52231919(70.84%)	5519806(7.49%)	26325861(35.7%)	25906058(35.13%)	26213746(35.55%)	26018173(35.29%)
HTM3_3	69629756	61779865(88.73%)	57373254(82.4%)	4406611(6.33%)	28907879(41.52%)	28465375(40.88%)	28672396(41.18%)	28700858(41.22%)
NTM3_1	71550762	54991562(76.86%)	49006688(68.49%)	5984874(8.36%)	24855364(34.74%)	24151324(33.75%)	24571559(34.34%)	24435129(34.15%)
NTM3_2	71748010	62047478(86.48%)	57391328(79.99%)	4656150(6.49%)	29028863(40.46%)	28362465(39.53%)	28626196(39.9%)	28765132(40.09%)
NTM3_3	71563032	54207504(75.75%)	48160707(67.3%)	6046797(8.45%)	24258761(33.9%)	23901946(33.4%)	24167051(33.77%)	23993656(33.53%)
HTM4_1	72875350	63305104(86.87%)	55538393(76.21%)	7766711(10.66%)	28082129(38.53%)	27456264(37.68%)	27839687(38.2%)	27698706(38.01%)
HTM4_2	70733800	53450081(75.57%)	46993043(66.44%)	6457038(9.13%)	23718639(33.53%)	23274404(32.9%)	23629102(33.41%)	23363941(33.03%)

HTM4_3	77221690	60471560(78.31%)	53944609(69.86%)	6526951(8.45%)	27223726(35.25%)	26720883(34.6%)	27108747(35.11%)	26835862(34.75%)
NTM4_1	70697556	54498703(77.09%)	48160314(68.12%)	6338389(8.97%)	24295549(34.37%)	23864765(33.76%)	24149321(34.16%)	24010993(33.96%)
NTM4_2	74513376	60784952(81.58%)	54816502(73.57%)	5968450(8.01%)	27676268(37.14%)	27140234(36.42%)	27367915(36.73%)	27448587(36.84%)
NTM4_3	74686518	65835297(88.15%)	60829867(81.45%)	5005430(6.7%)	30627632(41.01%)	30202235(40.44%)	30485361(40.82%)	30344506(40.63%)
HTM5_1	76301712	68223088(89.41%)	62882455(82.41%)	5340633(7.0%)	31658220(41.49%)	31224235(40.92%)	31431741(41.19%)	31450714(41.22%)
HTM5_2	71241782	62299522(87.45%)	55064230(77.29%)	7235292(10.16%)	27774677(38.99%)	27289553(38.31%)	27569193(38.7%)	27495037(38.59%)
HTM5_3	72130156	61510826(85.28%)	55250533(76.6%)	6260293(8.68%)	27885665(38.66%)	27364868(37.94%)	27721513(38.43%)	27529020(38.17%)
NTM5_1	69427786	57971935(83.5%)	52402240(75.48%)	5569695(8.02%)	26433700(38.07%)	25968540(37.4%)	26284079(37.86%)	26118161(37.62%)
NTM5_2	70824832	59994078(84.71%)	51894250(73.27%)	8099828(11.44%)	26258579(37.08%)	25635671(36.2%)	26002092(36.71%)	25892158(36.56%)
NTM5_3	71909312	60734638(84.46%)	54111161(75.25%)	6623477(9.21%)	27251633(37.9%)	26859528(37.35%)	27086466(37.67%)	27024695(37.58%)
